# Factors associated with complex surgical wounds in breast and abdomen: a case-control observational study

**DOI:** 10.1590/1518-8345.2274.3052

**Published:** 2018-10-11

**Authors:** Josimare Aparecida Otoni Spira, Eline Lima Borges, Patrícia Aparecida Barbosa Silva, Mery Natali Silva Abreu, Antônio Carlos Martins Guedes, José Ferreira Pires-Júnior

**Affiliations:** 1 Universidade Federal de Minas Gerais, Escola de Enfermagem, Belo Horizonte, MG, Brazil.; 2 Universidade Federal de Minas Gerais, Escola de Medicina, Belo Horizonte, MG, Brazil.; 3 Instituto Mario Penna, Hospital Luxemburgo, Belo Horizonte, MG, Brazil.

**Keywords:** Risk Factors, Protective Factors, Postoperative Period, Surgical Wound Dehiscence, Abdomen, Breast

## Abstract

**Objective:**

to identify factors associated with complex surgical wounds in the breasts and abdomen in outpatients.

**Method:**

observational case-control study involving 327 patients, distributed into 160 cases (complex surgical wound) and 167 controls (simple surgical wound). Data were extracted from the medical records and a binary logistic regression model was used for analysis, considering a significance level of 5%.

**Results:**

the factors associated with greater chance of occurrence of complex surgical wound were 18 to 59 years of age (p = 0.003), schooling < 8 years (p = 0.049), radiotherapy (p < 0.001), hysterectomy (p = 0.003), glycemia (≤ 99 mg/dL) and arterial hypertension (p = 0.033), while quadrantectomy (p = 0.025) served as a protective factor.

**Conclusion:**

radiotherapy was the most significant factor for surgical wound complications. Glycemic alteration was an unexpected result and shows the need for further studies related to this topic.

## Introduction

A surgical wound is characterized by an intentional rupture of the epithelial integrity of the skin and underlying structures. The ideal and most frequent healing process consists of the juxtaposition and alignment of the edges of the wound (first intention process), with the proliferative phase being completed in up to four weeks. In turn, second intention wound healing happens when the surgical wound remains open after the surgery and the proliferative phase occurs to repair a greater tissue loss. And finally, third intention healing happens when the surgical wound is left open for a short period of time and the re-approximation of the edges is performed later^1^. Wound healing for first intention, called surgical site, can become complex (complex surgical wound) when the suture opens as the result of local complications such as seroma, hematoma, infection and dehiscence, requiring a longer time for spontaneous closure^2^.

The prevalence of complex surgical wounds differs across countries according to the method of wound classification adopted in the studies and regional differences^3^, ranging from 0.21 per 1,000 people (95% confidence interval: 0.18-0.24)^4^ to 0.41 per 1,000 people (95% CI: 0.35-0.47)^3^ in the UK, 20.8% in the United States^5^ and 15.0% in Brazil^6^. As an aggravating factor to the epidemiological scenario of complex wounds, a recent study in the rural community of Rwanda, Africa, pointed to a probable underestimation of data due to the persistent inequities in access to surgical procedures, especially in low- and middle-income countries^7^. Epidemiological data regarding complex surgical wounds are scarce and the existing ones approach the subject in general way, without specifying the site of occurrence, as for example, in abdomen and breasts.

The growing increase in the number of cases of chronic wounds in the population, as described in a study carried out in Germany^8^, is also reflected in the financial burden and demand of human resources globally, representing an obstacle to health systems and to the economic development of each country. In addition, there are indirect costs, including economic loss for families, frustration for patients and caregivers, reduced quality of life, physical limitations, psychological disorders and social isolation^9^ ^-^ ^10^.

The biological aspects involved in the wound healing process is well documented^11^ ^-^ ^12^ and is based on four consecutive stages (hemostasis, inflammation, proliferation and tissue remodeling). Any change in the healing pathway may impair complete wound closure and cases of complications require special care. In contrast, the determination of risk factors that affect the healing process of surgical wounds is not yet clear in the literature^13^ ^-^ ^14^. Among the main causes of surgical wound dehiscence are local infection, flaws in the surgical technique, excessive stress at the edges of the wound, and low perfusion in the area. In turn, local risk factors for complication of surgical site constitute a traumatic process in the wound after surgeries and radiotherapy. Among the systemic risk factors are age, malnutrition, obesity, smoking, immunological impairment, associated consumptive diseases, chronic use of steroidal drugs or immunotherapeutic drugs. Emergency surgery also increases the chances of surgical wound dehiscence^15^.

In this context, the management of the treatment of surgical wounds should include the identification of possible critical factors for healing so as to provide individualized care. Nursing performance has an important role in monitoring the evolution of the wound healing process in general, however, previous studies^10^ ^,^ ^16^ ^-^ ^17^ have shown flaws and lack of knowledge in clinical practice, impacting the quality of care.

In view of the literary scope, while the biological and epidemiological aspects of chronic wounds, especially venous and arterial ulcers, diabetic foot and pressure injury have been extensively researched^9^, there is a lack of national and international information on complex surgical wounds, including guidelines directed at the prevention and treatment of these wounds.

Thus, in order to advance the knowledge on the theme, the objective of this study was to identify factors associated with complex surgical wounds in breasts and abdomen in outpatients.

## Method

This is an observational case-control study performed in a large tertiary university hospital in Belo Horizonte, Minas Gerais, Brazil.

The inclusion criteria for selecting the sample were patients of both sexes, aged 18 years or older, submitted to surgical procedures in the mammary and abdominal area from January 2003 to November 2015. Patients with prosthesis in the area of the breast, operative wounds submitted to the second intervention still in the immediate postoperative period, small incisions due to insertion of drains and tubes, and incomplete medical records in three or more variables of the study were excluded. After selecting the sample, after checking compliance with the aforementioned criteria, 327 patients were selected. Because the study consisted in a census of all the cases under study, sample calculations are not justified.

The participants were divided in two groups: *case group*, composed of 160 patients with complex surgical wounds developed within up to 30 days after the surgery, in the hospital or residential setting, with second intention wound healing; and the *control group*, composed of 167 patients with simple surgical wound with first intention wound healing within up to 30 days after surgery.

Simple surgical wound was the one that maintained apposed wound edges with the aid of the suture and presented complete epithelization within a period of 48 hours. Complex surgical wound was the one that had partial or total suture dehiscence, regardless of the cause, and demanded a time of more than four weeks for complete healing.

It should be emphasized that in this study a period of 30 days after the surgical procedure was delimited to analyze the outcome because it is during this time that patients are under the chance of developing surgical site infection, in the absence of a prosthesis implant. In the last case, infection may occur within a year after the surgical procedure^6^.

Data collection took place between December 2015 and June 2016 through a semi-structured questionnaire applied by two researchers responsible for the research. The information was extracted by consulting the patient’s medical records. Data regarding the preoperative period up to the 30^th^ postoperative day were included, regardless of whether the patient was hospitalized or was discharged.

The dependent variable consisted of surgical wounds healed by first intention (simple surgical wounds) in the *control group*, and surgical wounds healed by second intention (complex surgical wound) in the *case group*. The independent variables included sociodemographic variables (gender, age group categorized in adults [18-59 years old] and elderly [60 years and over], schooling, family income); behavioral variables (alcoholism, smoking); morbidities (neoplasia, arterial hypertension, diabetes mellitus); Body Mass Index (BMI) categorized according to literature-based cut-off points^18^ (low weight: < 18.5 kg/m^2^, eutrophy: 18.5 to 24.9 kg/m^2^, overweight: 25.0 to 29.9 kg/m^2^ and obesity: ≥ 30.0 ​​kg/m^2^), neoadjuvant treatment (chemotherapy, radiotherapy), serum biomarkers (albumin, hemoglobin, fasting serum glycemia). For the analysis of the results of serum biomarkers, values classified within the range of normality were considered, being the hemoglobin for men ≥13g/dL and for women ≥12g/dL, fasting serum glucose ≤99 mg/dL and Albumin with an interval between 3.5 and 5.2 g/dL. The variables related to the surgical wound consisted of topography (abdomen, breast) and type of surgical intervention. The variables chosen were treated in a previous study on complex surgical wounds, directed by the authors of this study^2^

Data analysis was performed in the *Statistical Package for Social Sciences* (SPSS, version 23.0, Chicago, IL, USA). Initially, a descriptive analysis was performed, expressed as proportions. Pearson’s chi-square test or Fisher’s exact test, when indicated, were used to compare the categorical variables. Afterwards, a multivariate analysis was performed using binary logistic regression with all the independent variables that presented p < 0.20 in the univariate analysis, and using the *Backward* method to remove the variables from the model. The variable neoplasia did not enter into the multivariate model due to the existence of collinearity with the variable radiotherapy. Thus the option was to include only the latter variable. To compose the final model, after adjusting for confounding factors, variables with p < 0.05 were considered statistically significant. The values ​​obtained were expressed as *odds ratio* (OR) and their respective 95% CI. The fit of the final model was evaluated by the *Hosmer & Lemeshow* test. We also tested the plausible interactions contained in the final model.

The project was approved by the Research Ethics Committee of the Federal University of Minas Gerais under Opinion nº 01978412.0.0000.5149. Due to the fact that this was a medical research, the researchers signed the Term of Commitment to use the data.

## Results

From the total of 327 patients submitted to breast or abdominal surgeries, 160 individuals presented complications in the surgical wound and composed the group of cases studied here, and the remaining (n = 167) patients who did not present complications composed the control group, in a 1: 1 ratio.

The sample consisted mainly of female patients (79.2%). The mean age was 51.4 (standard deviation = 15.3) years. Almost half (42.8%) of the participants did not complete elementary education, and 26.7% received one minimum wage. Regarding behavioral habits, 14.1% were alcoholics and 17.2% were smokers. Regarding the chronic conditions, neoplasia (67.6%), hypertension (41.9%) and diabetes mellitus (18.0%) prevailed. As for anthropometric data, 34.3% of the individuals were eutrophic. Regarding laboratory tests, serum albumin, hemoglobin and fasting blood glucose levels were altered in 36.6%, 35.5% and 42.4% of the sample, respectively. The most prevalent type of surgical intervention was mastectomy (30.6%), followed by quadrantectomy (sectorectomy) (11.6%). In 50.2% of the patients, the wounds were located in the abdominal region and the most used neoadjuvant treatment was chemotherapy (22.6%). Further details of the sample are given in Tables 1 and 2.

**Table 1:** Characteristics of the study sample according to demographic and behavioral variables in cases (complex surgical wound) and controls (simple surgical wound). University Hospital, Belo Horizonte, MG, Brazil, 2003 to 2015 (n = 327)

Variables	Total (n = 327)	Cases (n = 160)	Controls (n = 167)	p-value*	OR^†^ [95% CI]^‡^
	n (%)	n (%)	n (%)		
Sex					
Male	68 (20.8)	26 (38.2)	42 (61.8)		1.00 (ref.)
Female	259 (79.2)	134 (51.7)	125 (48.3)	0.047	1.73 [1.00-2.99]
Age group (years)					
18-59	221 (67.6)	117 (52.9)	104 (47.1)	0.036	1.65 [1.03-2.64]
≥ 60	106 (32.4)	43 (40.6)	63 (59.4)		1.00 (ref.)
Schooling (years)					
≥ 8	155 (57.2)	75 (48.4)	80 (51.6)		1.00 (ref.)
< 8	116 (42.8)	85 (73.3)	31 (26.7)	<0.001	2.93 [1.74-4.91]
Family Income (MW) §					
1	69 (26.7)	38 (55.1)	31 (44.9)		1.00 (ref.)
2-3	152 (58.9)	98 (64.5)	54 (35.5)	0.184	1.48 [0.83-2.64]
≥ 4	37 (14.3)	24 (64.9)	13 (35.1)	0.331	1.51 [0.66-3.44]
Alcoholism					
Non-alcoholic	245 (75.2)	123 (50.2)	122 (49.8)		1.00 (ref.)
Alcoholic	46 (14.1)	23 (50.0)	23 (50.0)	0.980	1.01 [0.54-1.89]
Ex-alcoholic	35 (10.7)	14 (40.0)	21 (60.0)	0.372	0.67 [0.27-1.62]
Smoking					
Non-smoker	237 (72.9)	114 (48.1)	123 (51.9)		1.00 (ref.)
Smoker	56 (17.2)	36 (64.3)	20 (35.7)	0.031	1.94 [1.06-3.55]
Ex-smoker	32 (9.8)	10 (31.3)	22 (68.8)	0.077	0.49 [0.22-1.08]

Variations in the total n are due to missing data. *p-value: differences in proportions (Chi-square test or Fisher’s exact test). †OR: Odds ratio. ‡ CI: Confidence Interval. §¶MW: minimum wage (Brazil) in the time of the study - R$ 240.00 (2003); R$ 260.00 (2004); R$ 300.00 (2005); R$ 350.00 (2006); R $380.00 (2007); R $415.00 (2008); R$ 465.00 (2009); R$ 510.00 (2010); R$ 540.00 and R$ 545.00 (2011); R$ 622.00 (2012); R$ 678.00 (2013); R$ 724.00 (2014); R$ 788.00.

**Table 2:** Characteristics of the study sample according to the clinical variables in the cases (complex surgical wound) and controls (simple surgical wound). University Hospital, Belo Horizonte, MG, Brazil, 2003 to 2015 (n = 327)

Variables	Total (n=327)	Cases (n = 160)	Controls (n = 167)	p-value*	OR^†^ [95% CI]^‡^
	n (%)	n (%)	n (%)		
Morbidities					
Neoplasms	221 (67.6)	100(45.2)	121 (54.8)	0.055	0.63 [0.40-1.01]
Arterial hypertension	137 (41.9)	73 (53.3)	64 (46.7)	0.181	1.35 [0.87-2.10]
Diabetes mellitus	59 (18.0)	27 (45.8)	32 (54.2)	0.591	0.86 [0.49-1.51]
BMI (kg/m^2^)^§^					
<18.5	22 (6.9)	11 (50.0)	11 (50.0)	0.531	0.29 [-0.62-1.21]
18.5 - 24.9	110 (34.3)	47 (42.7)	63 (57.3)		1.00 (ref.)
25.0 - 29.9	88 (27.4)	46 (52.3)	42 (47.7)	0.182	0.38 [-0.18-0.95]
≥ 30.0	101 (31.5)	52 (51.5)	49 (48.5)	0.203	0.35 [-0.19-0.90]
Albumin (g/dL)					
<3.5	59 (36.6)	32 (54.2)	27 (45.8)		1.00 (ref.)
≥ 3.5	102 (63.4)	54 (52.9)	48 (47.1)	0.874	0.95 [0.50-1.81]
Hemoglobin (g/%)					
≥ 12 (F)** and ≥ 13 (M)^††^	169 (64.5)	66 (39.1)	103 (60.9)		1.00 (ref.)
<12 (F)** and <13 (M)^††^	93 (35.5)	46 (49.5)	47 (50.5)	0.103	1.53 [0.92-2.55]
Fasting glycemia (mg/dL)					
> 99	100 (42.4)	34 (34.0)	66 (66.0)		1.00 (ref.)
≤ 99	136 (57.6)	64 (47.1)	72 (52.9)	0.044	1.73 [1.01-2.94]
Wound topography					
Abdomen	164 (50.2)	82 (50.0)	82 (50.0)		1.00 (ref.)
Breast	163 (49.8)	78 (47.9)	85 (52.1)	0.698	0.92 [0.60-1.42]
Surgical intervention					
Mastectomy	100 (30.6)	50 (50.0)	50 (50.0)	0.797	1.06 [0.66-1.70]
Hysterectomy	22 (6.7)	21 (95.5)	1 (4.5)	<0.001	25.08 [3.33-188.81]
Laparotomy	22 (6.7)	17 (77.3)	5 (22.7)	0.006	3.85 [1.39-10.71]
Hernioplasty	15 (4.6)	13 (86.7)	2 (13.3)	0.003	7.30 [1.62-32.87]
Surgical drainage	12 (3.7)	11 (91.7)	1 (8.3)	0.003	12.26 [1.56-96.06]
Quadrantectomy	38 (11.6)	7 (18.4)	31 (81.6)	<0.	0.20 [0.09-0.47]
Exeresis	6 (1.8)	6 (100.0)	0 (0)	0.013	^‡‡^
Amputation of the rectum	8 (2.4)	6 (75.0)	2 (25.0)	0.166	3.21 [0.64-16.17]
Cesarean section	6 (1.8)	6 (100.0)	0 (0)	0.013	^‡‡^
Colectomy	13 (4.0)	5 (38.5)	8 (61.5)	0.441	0.64 [0.21-2.00]
Neo-adjuvant					
Chemotherapy	74 (22.6)	37 (50.0)	37 (50.0)	0.834	1.06 [0.63-1.77]
Radiotherapy	50 (15.3)	45 (90.0)	5 (10.0)	<0.001	12.68 [4.88-32.93]

Variations in the total n are due to missing data. *p-value: differences in proportions (Chi-square test or Fisher’s exact test). †OR: Odds ratio. ‡CI: Confidence Interval. BMI: Body mass index. **F: Female. ††M: Male. ‡‡ It was not possible to calculate OR due to the presence of null cases.

The independent variables that presented the greatest chance for surgical wound complications (p < 0.05) in the univariate analysis were female gender (OR = 1.73, 95% CI = 1.00-2.99), age group 18-59 years (OR = 1.65; 95% CI = 1.03-2.64), schooling < 8 years (OR = 2.93; 95% CI = 1.74-4.91), smoking habit (OR = 1.94; 95% CI = 1.06-3.55), fasting glycemia ≤ 99 mg/dL (OR = 1.73; 95% CI = 1.01-2.94), hysterectomy surgery (OR = 25.08; 95% CI = 3.33-188.81), laparotomy (OR = 3.85; 95% CI = 1.39-10.71), hernioplasty (OR = 7.30; 95% CI = 1.62-32.87), surgical drainage (OR = 12.26; 95% CI = 1.56-96.06) and radiotherapy (OR = 12.68; 95% CI = 4.88-32.93). The surgical procedures exeresis and cesarean section also acted as factors that increased the risk for complex surgical wound; however, it was not possible to calculate the risk (OR) due to the existence of null cases. On the other hand, quadrantectomy (sectorectomy) (OR = 0.20; 95% CI = 0.09-0.47) was associated with a lower chance of a complex surgical wound (Tables 1 and 2).

After controlling for the confounding variables through logistic regression analysis, the following variables remained significantly associated (p < 0.05) with a greater chance of occurrence of the outcome (complex surgical wound): radiotherapy (OR = 36.13; 95% CI = 8.47-154.11), hysterectomy surgery (OR = 13.34; 95% CI = 1.59-112.00), schooling < 8 years (OR = 2.28; 95% CI = 1.00-5.20), hernioplasty (OR = 36.41; 95% CI = 3.35-395.06), laparotomy (OR = 7.80; 95% CI = 1.91-31.91), age group 18-59 years (OR = 4.58; 95% CI = 1.69-12.40), fasting glycemia ≤ 99mg/dL (OR = 3.31; 95% CI = 1.39-7.88) and arterial hypertension (OR = 2.65; 95% CI = 1.08-6.50). In the multivariate analysis, quadrantectomy (sectorectomy) (OR = 0.08; 95% CI = 0.008-0.72) remained a factor associated with a lower chance of occurrence of complex surgical wound (Table 3). The adjustment test (Hosmer & Lemeshow test) of the final model was satisfactory (p-value = 0.2946). There was no interaction between covariables in the final model (Table 3).

**Table 3:** Factors associated with the presence of complex surgical wound (cases) and simple surgical wound (controls). University Hospital, Belo Horizonte, MG, Brazil, 2003 to 2015 (n = 327)

Variables	OR_ajusted_*	95% CI^†^	p-value^‡^
Neoadjuvant/Radiotherapy			
No	1.00 (ref.)		
Yes	36.13	8.47-154.11	<0.001
Hysterectomy			
No	1.00 (ref.)		
Yes	13.34	1.59-112.00	0.017
Schooling (years)			
≥ 8	1.00 (ref.)		
< 8	2.28	1.00-5.20	0.049
Quadrantectomy			
No	1.00 (ref.)		
Yes	0.08	0.008-0.72	0.025
Hernioplasty			
No	1.00 (ref.)		
Yes	36.41	3.35-395.06	0.003
Laparotomy			
No	1.00 (ref.)		
Yes	7.80	1.91-31.91	0.004
Age group (years)			
18-59	4.58	1.69-12.40	0.003
≥ 60	1.00 (ref.)		
Fasting glycemia (mg/dL)			
> 99	1.00 (ref.)		
≤ 99	3.31	1.39-7.88	0.007
Arterial hypertension			
No	1.00 (ref.)		
Yes	2.65	1.08-6.50	0.033

*OR: Odds ratio. †CI: Confidence Interval. ‡p-value: differences in proportions (logistic regression).

## Discussion

Surgical wound complications may lead to longer hospitalization, readmissions, and multiple surgeries^3^, with an estimated cost of $6.5 billion in the United States^15^.

The identification of the risk factors for surgical wound complication, as well as a better understanding of the changes in the wound healing process induced by causative agents allows improvements in healing process outcomes. It is worth noting that multiple local and systemic risk factors are common and act independently or, concomitantly, potentiating the synergic effect of the deleterious action on the wound healing process^13^.

Among the nine independent variables kept in the multivariate model, four referred to the type of surgical intervention. Similar results were identified in previous studies^19^ ^-^ ^21^.

An epidemiological study with a sample of 175 patients submitted to gynecological surgeries reported an incidence of 10.9% of complications in the postoperative period, being surgical site infection the most frequent. It was noted that 19.8% of the surgeries were hysterectomies^20^. In another study aimed at identifying postoperative complications in patients submitted to hysterectomy, sepsis due to hematoma of the surgical wound was identified in four patients among 98 submitted to abdominal hysterectomy^22^. The routes for performing procedures involving the uterus and appendages are the vaginal, abdominal and laparoscopic routes, and the abdominal approach is most frequent in some centers^20^.

Laparotomy as a risk factor for development of complex surgical wounds was confirmed in other studies^19^ ^,^ ^23^. Such studies also identified a highest incidence of surgical wound dehiscence to be related with emergency laparotomy, duration of the surgical procedure of more than two hours, abdominal distension, potential contamination, and surgical site infection, the latter being the most common factor.

In a study involving 476 patients submitted to hernioplasty with mesh, 6.5% developed deep surgical site infection, with surgery time being the only significant associated risk factor^24^. However, in another study involving 146 patients submitted to hernioplasty surgery with mesh, among 73 open surgeries and another 73 by laparoscopy, it was observed that 31% of the patients who underwent the laparoscopic procedure had surgical site infection, compared with 48% of the others who had open surgeries^21^.

In turn, patients who underwent a quadrantectomy had a lower chance of developing a complex surgical wound. Breast cancer treatment involves two main types of surgery: conservative breast surgery and mastectomy. The first one, also called quadrantectomy, tumorectomy, partial mastectomy or segmental mastectomy, consists of the removal of the segment or sector of the breast that contains the tumor. The goal is to remove the tumor along with some normal adjacent tissue. On the other hand, total mastectomy consists of the extraction of the breast, including all breast tissue and sometimes other nearby tissues^25^. Quadrantectomy is obviously less aggressive when compared to other surgical approaches, including those cited in this study where patients were more likely to present surgical wound complications.

Associated diseases and treatments may also influence the rate of surgical wound complications. In the present study, patients with arterial hypertension and those who had undergone neoadjuvant radiotherapy had a higher chance of complications. Hypertension is a chronic disease capable of altering normal blood flow, culminating in decreased supply of oxygen and nutrients necessary for the healing of tissues that are damaged in the surgical procedure^26^. Although hypoxia attracts the displacement of neutrophils and macrophages, oxygen is important to the healing process because it favors phagocytosis. It is also essential for the deposition of collagen, acting as a substrate in the hydroxylation of proline and lysine residues^12^. When this process does not occur, the surgical wound may present complications, as evidenced in a study performed with patients undergoing laparotomy^27^. Arterial hypertension was associated with a higher probability of occurrence of surgical site infection with early postoperative dehiscence, as well as hypoproteinemia and malignant diseases^27^. Considering that hypertension is a modifiable risk factor, collective actions to prevent this chronic condition are necessary in order to reduce the complications resulting from high blood pressure levels. Despite the high prevalence of hypertension (approximately 600 million people)^28^, the literature on this disease as a risk factor for the development of complex surgical wound is scarce.

The predictive factor associated to complex surgical wound of greater statistical significance was neoadjuvant radiotherapy. This is a common treatment against cancers. However one of the undesirable effects of exposure to radiation is the occurrence of involuntary lesions in the underlying skin, causing tissue ischemia and in some cases ulcerations, consequently slowing the healing process of the wound^12^. The mechanisms responsible for these changes are complex and the existing treatment is limited. In order to elucidate this complexity, a systematic review was carried out with a sample of 93 articles. Prominent theories include cell depletion, stromal cell dysfunction, aberrant collagen deposition, and microvascular damage. Pro-inflammatory cytokines and free-radical cascades contribute to chronic radiation damage^29^.

However, the time between the end of the radiotherapy and the surgery seems to influence the appearance of the complications. A study^30^ performed with 511 patients submitted to abdominal wall reconstruction, of which 381 did not receive previous radiotherapy and 130 received, found no difference between groups in the rate of surgical wound complication. Another study carried out with 199 patients submitted to breast reconstruction; 100 patients had previously been irradiated and the remainder had not been irradiated. The study demonstrated that the irradiated patients had more complications such as surgical wound dehiscence and surgical site infection^31^. In both studies^30^ ^-^ ^31^, patients had complied with the three-week period of radiotherapy cessation in the preoperative period.

In the present study, schooling of less than eight years was also a risk factor for surgical wound complication. The importance and influence of the participation of patients in the prevention of infections are considered paramount for improving patients’ safety. The training of this public through provision of information necessary for their participation in the prevention of surgical site infection can play an important role in the implementation of recommendations and, and this is influenced by their level of schooling of the patients^32^. Another data related to the prevention of complications in the postoperative period is related to the adherence of patients to the guidelines given by the health team. The level of education of the patients can interfere in the learning process. This is therefore a factor that deserves attention in the health education process, because individuals with low instructional level demand more detailed information regarding forms of preventing infection.

Fasting glycemia of less than 99 mg/dL, found in this study as a risk factor for complex surgical wound, contradicts the literature where hyperglycemia appears as a factor that induces complications of complex wounds^11^ ^,^ ^33^. However, hyperglycemia is present in as a response to surgical trauma. Surgical aggression triggers a neuroendocrine and metabolic reaction with changes that translate into a hypercatabolic state, with elevated plasma levels of catabolic hormones (cortisol, glucagon, catecholamines) and hormone release by the hypothalamus stimulating the release of adrenocorticotrophic hormone, growth hormone, prolactin, endorphins and antidiuretic hormone by the pituitary. This process presents two distinct phases of metabolic response to trauma: an early phase and a late phase. The first phase, lasting two to three days, occurs immediately after the aggression and is characterized by hemodynamic instability, represented by hypovolemia, hypotension, decreased blood flow, increased systemic vascular resistance, and increased insulin, catecholamines, and circulating glucocorticoids and mineralocorticoids. The second phase is the hyperdynamic response to aggression, symbolized by water retention, increased vascular permeability, decreased vascular resistance, with increasing catecholamines, glucocorticoids, producing hyperglycemia and proteolysis, the common denominator being hypermetabolism. The two phases have hyperglycemia in common. Normally, after surgical stress, most patients recover their major vital functions within four to five days^34^. Furthermore, diabetes mellitus is responsible for microvascular lesions that affect tissue oxygen levels and nutrient supplies; thus, high glycemic levels also affect leukocyte function, essential in the wound healing pathway^12^.

Another contradiction identified in this study concerns the greater susceptibility of surgical wound complication in people aged between 18 and 59 years. However, this result is not unanimous among researchers^19^ ^,^ ^23^. Previous study showed that patients over 80 years of age presented a higher risk for wound dehiscence, besides a significantly increased risk of second surgery due to superficial infection or bleeding^35^. With advancing age, the epidermal layer tends to become thinner and the inflammatory, migratory and proliferation responses slower. These patients are also more prone to multiple chronic conditions and to a greater number of associated risk factors, potentiating deleterious action on wound healing pathways^12^ ^-^ ^13^.

From another perspective, the impact of the consequences of complex surgical wounds on younger people tends to be more serious compared to the elderly as a result of the greater probability that people in younger age groups are inserted in the labor market, providing the support for their families. This assertion is supported by a recent study^10^ that evaluated the perceptions of people living with complex surgical wounds, reinforcing the uncertainties and financial constraints due to the limitations imposed by non-healing of the wound, loss of employment and interference with the life cycle of the family.

In addition to the findings of this study, probable underestimation of risk factors should be considered as a result of the fact that the data was extracted from a secondary source (medical records). Also, an interesting study^7^ conducted in the rural community of Rwanda, Africa, showed a prevalence of 12.0% (95% CI: 9.2-14.9) of untreated surgical conditions due to lack of access to the surgical procedure, with 5.3% (95% CI: 3.3-7.3) of the conditions caused by lesions/wounds. Therefore, our findings are attributable to those people who had access to surgical procedures, excluding people who were at home without the opportunity for treatment. This reality is worrying because estimates indicate that there are approximately 5 billion people in the world without access to surgical care when necessary, a consequence of social inequities in access to treatment^36^. This may result in the worsening of the patient’s clinical condition, requiring emergency surgeries and implying a greater chance of complications in the healing process of the postoperative wound.

Initiative for better management of chronic wound care has been proposed in Australia with the creation of a *Mobile Wound Care* (MWC) database. The MWC is an electronic web-based system of chronic wound care in the rural community of the Gippsland region, containing information on wound etiology, healing process and treatment costs^13^. Other programs have been developed in countries with social problems such as in Haiti and in African countries, evidencing improvements in the indicators of control of complications of complex wounds^36^.

Thus, the nursing team should assume responsibility for patients safety, so that its clinical decisions be in line with the best scientific evidence available. However, there are records in the literature of failures in the nursing care process, as evidenced in previous research^10^ ^,^ ^16^ ^-^ ^17^. It is possible to mention the dissatisfaction of patients with complex surgical wounds with the lack of continuity and quality of nursing care in the management of the complex wound, particularly the lack of information about the care to be provided to the wound in post-discharge moments, conflicting pieces of information provided by different healthcare professionals and failure to comply with the frequency of exchange of dressings at home^10^.

Strict knowledge of risk factors for surgical wound complications in breast and abdominal regions contributes to better systematization of care and planning of therapeutic strategies, including greater caution for certain types of surgery, as well as, chronic conditions, exposure to radiotherapy, instructional level and possible social vulnerability for people at productive age. Furthermore, although the present study did not identify an association between obesity and smoking, previous research has shown that weight reduction^13^ and smoking cessation^37^ favor the healing process of the wound. In this sense, care must be based on a holistic, patient-centered approach^13^.

One of the limitations of this study is the fact that the data came from a single hospital institution, which is a reference in the care of complex wounds. Due to this, the data should be interpreted with caution. Another possible limitation is associated to the reliability of notes in medical records and retrospective data, with the possibility of information bias. These facts made it impossible to identify the cause of surgical wound complication, whether it was hematoma, infection or dehiscence.

Data from a secondary source may have influenced the outcome related to fasting glycemia of less than 99 mg/dL as a risk factor for complex surgical wound. However, the weaknesses cited do not lessen the relevance of the study because of its contribution to new knowledge on the theme.

This study aims to contribute to a better understanding of the factors related to surgical wound complications. The results obtained support the nursing care to patients who undergo surgical procedures, especially those involving the abdomen and breasts. In addition, they will support the creation or revision of specific protocols, aiming to reduce the occurrence of complications in these wounds and, consequently, their economic and biopsychosocial impacts.

The adequate identification of risk factors for surgical wound complications implies the improvement of care processes and the reduction of these postoperative complications. Given the scarce literature on the subject, it is necessary to invest more in scientific productions, with a design of more robust clinical studies and relevant actions of continuing education for people inserted in the health area.

## Conclusion

In short, this study identified radiotherapy as a factor of greater significance for surgical wound complications. Other associated factors were the type of surgical intervention, chronic condition, radiation exposure, instructional level and age. Quadrantectomy, which is a less invasive surgery, acted as a protective factor. In contrast, fasting glycemia below 99 mg/dL appeared as a risk factor; this was an unexpected result showing the need for a better understanding of the glycemic effects on the wound healing process.

Safety flaws in care processes, added to extrinsic and intrinsic characteristics of the subjects, can result in significant complications in the healing of surgical wounds. Previous Knowledge of the factors that trigger these complications is extremely important to plan preventive actions.
